# Preface to the theme issue ‘Physics-informed machine learning and its structural integrity applications (Part 2)’

**DOI:** 10.1098/rsta.2023.0248

**Published:** 2024-01-08

**Authors:** Shun-Peng Zhu, Abílio M. P. De Jesus, Filippo Berto, John G. Michopoulos, Francesco Iacoviello, Qingyuan Wang

**Affiliations:** ^1^ School of Mechanical and Electrical Engineering, University of Electronic Science and Technology of China, Chengdu 611731, People's Republic of China; ^2^ INEGI, Faculty of Engineering, University of Porto, Porto 4200-465, Portugal; ^3^ Department of Chemical Engineering, Materials and Environment, Sapienza University of Rome, 00184 Roma, Italy; ^4^ Computational Multiphysics Systems Lab, Center for Materials Physics and Technology, Naval Research Laboratory, Washington, District of Columbia, USA; ^5^ Department of Civil and Mechanical Engineering, University of Cassino and Southern Lazio, Cassino, Italy; ^6^ MOE Key Laboratory of Deep Earth Science and Engineering, College of Architecture and Environment, Sichuan University, Chengdu 610065, People's Republic of China; ^7^ Advanced Research Institute, Chengdu University, Chengdu 610106, People's Republic of China

**Keywords:** machine learning, physics-informed machine learning, structural integrity, failure mechanism modelling, prognostic and health management

## Abstract

As an emerging research field, physics-informed machine learning and its structural integrity applications may bring new opportunities to the intelligent solution of engineering problems. Pure data-driven approaches have some limitations when solving engineering problems due to lack of interpretability and data hungry applications. Therefore, further unlocking the potential of machine learning will be an important research direction in the future. Knowledge-driven machine learning methods may have a profound impact on future engineering research. The theme of this special issue focuses on more specific physics-informed machine learning methods and case studies. This issue presents a series of practical ideas to demonstrate the huge potential of physics-informed machine learning for solving engineering problems with high precision and efficiency.

This article is part of the theme issue ‘Physics-informed machine learning and its structural integrity applications (Part 2)’.

Structural integrity and safety are key to ensuring reliable operation of engineering systems/facilities. The analysis and modelling of engineering problems (such as fatigue performance prediction, health monitoring, reliability assessment) have roughly gone through the following stages: empirical science based on induction and summary, computational science based on mathematical derivation, computational science based on data-driven and information science that are mainly information-driven. Obviously, machine learning, as a typical representative of data-driven methodologies (almost equal), can provide efficient and accurate solutions to engineering problems. In this issue, the reader will find active learning-based structural reliability analysis, data-driven-based fatigue life prediction and notched component fracture prediction.

However, as a black box model, the application of machine learning in engineering is still questionable. Meanwhile, the data-hungry nature of machine learning also greatly limits its development in structural integrity. Knowledge-driven approaches involve a broader context. There is no doubt that data contains physical information and machine learning also learns as much information as possible from it (it may also learn noise). Moreover, machine learning methods that integrate more diverse physical information have brought novel solutions to engineering issues. Introducing physical phenomena, physical laws and theoretical knowledge, that is, information, into machine learning models ensures that intelligent solving of engineering problems are physically consistent.

Accordingly, while discussing the application of physics-informed machine learning models in structural integrity, this special issue also exposes more specific knowledge-driven methods and case studies, such as physically informed-driven structural vibration identification, turbine cavitation condition monitoring and defect size estimation. It provides practical ideas to open up the mystery of machine learning, that is ‘interpretability’.

We believe that this issue will provide concrete ideas to academia and industry. Meanwhile, we also hope that its content can be applied to a wider range of engineering problems.

## Data Availability

This article has no additional data.

